# Investigating Domain-Specific Cognitive Impairment Among Patients With Multiple Sclerosis Using Touchscreen Cognitive Testing in Routine Clinical Care

**DOI:** 10.3389/fneur.2018.00331

**Published:** 2018-05-11

**Authors:** Jack Cotter, Nethmi Vithanage, Shuna Colville, Dawn Lyle, Denise Cranley, Francesca Cormack, Jennifer H. Barnett, Katy Murray, Suvankar Pal

**Affiliations:** ^1^Cambridge Cognition, Cambridge, United Kingdom; ^2^College of Medicine and Veterinary Medicine, University of Edinburgh, Edinburgh, United Kingdom; ^3^Anne Rowling Regenerative Neurology Clinic, University of Edinburgh, Edinburgh, United Kingdom; ^4^Department of Psychiatry, University of Cambridge, Cambridge, United Kingdom; ^5^Forth Valley Royal Hospital, Larbert, United Kingdom; ^6^Centre for Clinical Brain Sciences, University of Edinburgh, Edinburgh, United Kingdom

**Keywords:** multiple sclerosis, cognition, Cambridge Neuropsychological Test Automated Battery, computerized testing, neuropsychological assessment

## Abstract

Cognitive dysfunction is present in up to 70% of patients with multiple sclerosis (MS) and has been reported at all stages and in all subtypes of the disease. These deficits have been reported across a variety of cognitive domains, but are generally under-recognized and incompletely evaluated in routine clinical practice. The aim of this study was to investigate the spectrum of cognitive impairment in patients with MS presenting to a specialist MS clinic using the Cambridge Neuropsychological Test Automated Battery (CANTAB), administered on a touchscreen platform. Ninety MS patients completed computerized CANTAB tasks assessing working memory, executive function, processing speed, attention, and episodic memory. Scores were adjusted for age, sex, and level of education and classified as normal or impaired based on comparison with a large normative data pool. We also investigated the impact of clinical and demographic variables which could potentially influence cognitive performance including patient educational level (a proxy for cognitive reserve), disease status (duration, course, and severity of MS), and depression. CANTAB testing detected cognitive impairment in 40 patients (44% of the sample). The most frequently impaired domain was executive function, present in 55% of cognitively impaired individuals. Disease duration and severity were significantly associated with performance across various cognitive domains. Patients with depressive symptoms were also more likely to exhibit impaired processing speed. Results from this study confirm that cognitive impairment is common and occurs across a range of domains among MS patients attending routine clinical visits. CANTAB tasks provide a sensitive and practical approach to cognitive testing in MS patients as part of a holistic patient assessment.

## Introduction

Cognitive dysfunction is present in up to 70% of patients with multiple sclerosis (MS) and has been reported at all stages and in all subtypes of the disease (1–3). These deficits have been reported across a variety of cognitive domains and are thought to arise as a result of the diffuse white and gray matter damage associated with MS (4–6). Cognitive dysfunction is a leading cause of disability in MS and is associated with unemployment (7, 8), increased caregiver burden (9, 10), and poor quality of life (11–13). However, cognitive impairment is often under-recognized and incompletely evaluated in patients with MS (6).

Cognitive deterioration is often overlooked, in part, due to a lack of tools suitable for use in routine clinical practice. Cognitive batteries are typically time-consuming, require specialist resources and a trained rater to administer and interpret the results of these tests. While a number of cognitive batteries have been developed specifically for use in patients with MS, including the Brief Repeatable Battery of Neuropsychological tests (BRB-N) (14), the Minimal Assessment of Cognitive Function in MS (15), and the Brief International Cognitive Assessment for MS (BICAMS) (16), these retain many of these practical limitations. There is a need for sensitive as well as time and resource-efficient tests for detecting cognitive impairment as part of routine holistic patient assessment in MS clinics.

The Cambridge Neuropsychological Test Automated Battery (CANTAB) consists of a number of computerized tests that can be administered *via* touchscreen platforms to assess discrete cognitive subdomains (17, 18). These have been developed and validated over the past 30 years and offer potential benefits over conventional paper-and-pencil based cognitive tests (19). These touchscreen tasks ensure accurate response scoring and provide precise assessment of reaction-time based measures. Automated instructions are available in a range of languages and tasks can be largely self-administered by patients, requiring no technical knowledge or prior familiarity. Automatic scoring and comparison to normative data also provides clinicians with an indication of a patient’s level of cognitive performance relative to individuals of a similar age, sex, and level of education in the general population. CANTAB tasks have demonstrated sensitivity to cognitive dysfunction and have been widely used across a range of clinical groups (20–23), including patients with MS (24–26). The aim of this study was to investigate domain-specific cognitive impairment in patients with MS using CANTAB in routine clinical care. We also sought to examine whether patient educational level [a proxy for cognitive reserve (27)], disease status (duration, course, and severity of MS), or depression were associated with cognitive performance.

## Materials and Methods

### Patients

105 consecutive patients attending a specialist MS clinic at the Anne Rowling Regenerative Neurology Clinic, University of Edinburgh, between March 2014 and October 2014 were offered the opportunity to complete a series of cognitive tests as part of their routine holistic clinical assessment. All patients included in the current study met McDonald criteria for MS (28). None of the patients were in acute relapse at the time of the assessment. Electronic patient records, clinical case notes, and patient interviews were reviewed to determine patient demographics, disease course, duration, and Expanded Disability Status Scale (EDSS) (29) score (reflecting the degree of physical disability). Patients were also screened for depression using a touchscreen version of the 15-item Geriatric Depression Scale (GDS) (30).

### Cognitive Testing

Three automated CANTAB neuropsychological tests were administered using a touchscreen tablet computer (iPad Air, Apple Inc.). The user-friendly touchscreen platform used for patient testing required no prior neuropsychological training for administration. Each task incorporated an automated computer voiceover providing instructions to patients. The cognitive assessment battery took approximately 15 minutes to complete:

1. Match to Sample: assesses processing speed and attention
2. Paired Associates Learning: assesses visuospatial episodic memory
3. Spatial Working Memory: assesses working memory and executive function (specifically strategic planning)

Upon completion of cognitive testing, patient raw scores for each cognitive domain were internally processed by the software to create *z*-scores. These *z*-scores were standardized for age, sex, and education status using data from an inbuilt normative database collected from healthy adults aged 18–90 years. Based on these *z*-scores, cognitive performance levels were categorized from “superior” (*z*-score > 1.5) to “very poor” (*z*-score < −2), though “average” (*z*-score ≥ −1) was the highest possible category for some tests. Patients scoring “poor” or “very poor” (*z*-score < −1.5) in a cognitive domain were considered to be impaired relative to their age, sex, and education-matched peers.

### Statistical Analyses

Data were analyzed using SPSS statistical software (IBM SPSS Statistics for Windows, Version 23.0. Armonk, NY, USA: IBM Corp.). Chi-square test and Fisher’s exact test were used to examine group differences in the proportion of patients demonstrating cognitive impairment in each cognitive domain based on level of education, depression, and MS disease course. Spearman’s rank-order correlation test was used to examine associations between disease duration and severity with cognitive performance, based on the aforementioned *z*-score derived performance categories. All analyses were two-tailed, *p* < 0.05 was considered statistically significant.

### Ethical Approval

As cognitive assessments were performed as part of routine clinical evaluation of patients, and clinical data were handled in an anonymized fashion, the Local Research Ethics Committee deemed that specific ethical approval was not required for this study.

## Results

### Patient Characteristics

**Figure 1:**
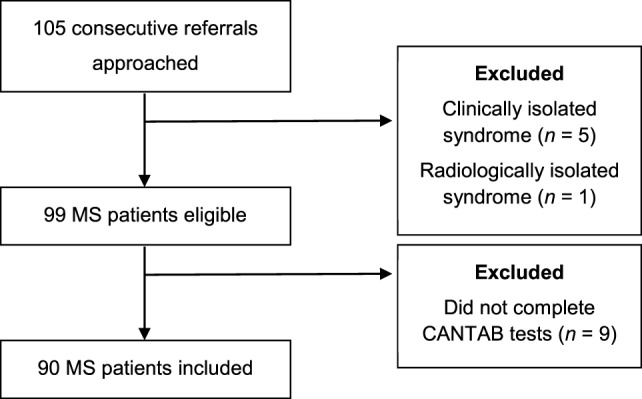
Patient recruitment flowchart.

Ninety MS patients completed the CANTAB assessments (mean age 44.5 ± 10.9 years, range: 22–70 years). The average duration of disease was 11.4 ± 9.0 years (range: 0–39 years). The average EDSS score was 3.6 ± 2.4 (range: 0–7.5). Further demographic and clinical characteristics of the patients included in this study are presented in Table 1. Fifteen patients were excluded (Figure 1), though further details on the MS patients that did not complete the CANTAB assessments (*n* = 9) were not recorded.

**Table 1:** Patient clinical and demographic characteristics.

		*n* (%)
Sex	Male	22 (24%)
	Female	68 (76%)
Level of education	Left before age 16	9 (10%)
	Left age 16–18	41 (46%)
	Left after age 18	40 (44%)
Multiple sclerosis disease course	Relapsing–remitting	65 (72%)
	Primary progressive	10 (11%)
	Secondary progressive	15 (17%)

### Cognitive Impairment in MS

**Figure 2:**
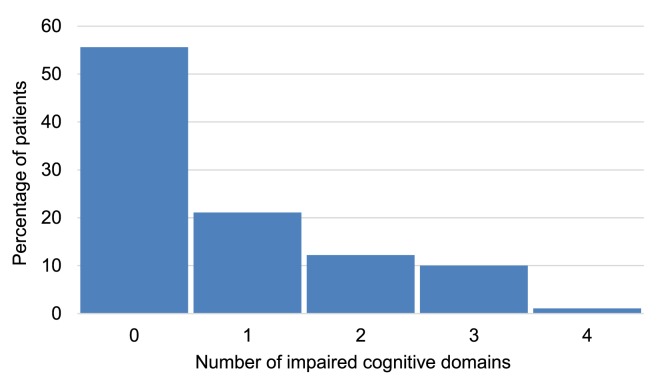
Percentage of multiple sclerosis patients with impairment in different cognitive domains.

**Figure 3:**
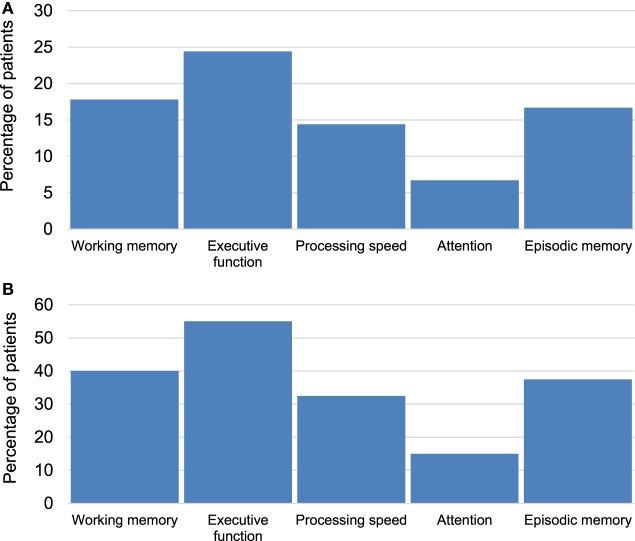
**(A)** Percentage of multiple sclerosis (MS) patients with impairment in specific cognitive domains across the whole sample (*n* = 90). **(B)** Percentage of MS patients with impairment in specific cognitive domains among the impaired group (*n* = 40).

CANTAB tests detected cognitive impairment in at least one cognitive domain among 40 (44%) of the patients. Twenty-three percent of cases demonstrated impairment across multiple cognitive domains (Figure 2). Executive function was the most commonly impaired domain (Figure 3A) and was present in 55% of patients who exhibited impairment in at least one cognitive domain (Figure 3B).

### Association With Education

For the purposes of the normative comparisons, the education level of patients was recorded into one of three categories; (1) those who left school before the age of 16, (2) those who left between ages 16 and 18, and (3) those who completed education after 18 years of age. As there were only a small number of individuals who had only completed up to 16 years of education (*n* = 9), we merged categories one and two for the purposes of examining whether cognitive impairment was associated with level of education across the sample. This was intended to serve as a proxy for cognitive reserve, given that higher levels of education are thought to be protective against cognitive decline in MS. We therefore conducted our analyses based on those individuals with ≤18 (*n* = 50) or >18 years of education (*n* = 40). There were no significant differences in the proportion of patients with cognitive impairment in any cognitive domain between these two groups (all *p* ≥ 0.22).

### Association With MS Disease Duration, Severity, and Course

Spearman’s rank-order correlations were performed to examine the associations between disease duration and severity with cognitive performance (Table 2). A longer disease duration was associated with poorer performance on measures of processing speed and attention. More severe physical disability (as assessed using the EDSS) was significantly associated with poorer performance on measures of working memory, executive function, processing speed, and attention. A higher proportion of patients with primary progressive (20%) and secondary progressive MS (13%) exhibited impaired attention compared to those with relapsing–remitting MS (3%), though this did not reach statistical significance (*p* = 0.056). There were no other statistically significant differences in the proportion of patients with impairment in any of the cognitive domains between the different MS disease courses (all *p* ≥ 0.38).

**Table 2:** Correlations between disease duration, severity, and cognitive performance.

	Disease duration (years)		Disease severity (Expanded Disability Status Scale score)	
		
	*r*_s_	*p*-Value	*r*_s_	*p*-Value
Working memory	−0.118	0.269	−0.215	0.042
Executive function	−0.083	0.436	−0.224	0.034
Processing speed	−0.218	0.039	−0.298	0.004
Attention	−0.206	0.051	−0.263	0.012
Episodic memory	−0.073	0.492	−0.132	0.216

### Association With Depression

Clinically significant depressive symptoms (GDS ≥ 5) were identified in 33 (37%) patients. One patient did not complete the GDS questionnaire and was, therefore, excluded from this analysis. When analyzed using Fisher’s exact test, a higher proportion of patients with depression exhibited impaired processing speed compared to non-depressed patients (*p* = 0.013). There were no significant differences in the proportion of cognitively impaired individuals between the depressed and non-depressed patients in any other domains (all *p* ≥ 0.061).

## Discussion

### Summary of Results

Cognitive impairment was identified using CANTAB in 44% of patients attending routine appointments at a specialist MS clinic. This figure is comparable to the proportion of MS patients affected by cognitive impairment reported in other studies based on longer and more resource-intensive cognitive batteries (31–33). Executive function (specifically strategic planning) was the domain most commonly impaired among patients. There was also evidence that both disease duration and degree of physical disability may be associated with the severity of cognitive dysfunction observed in patients with MS. These results demonstrate that CANTAB tasks can be used to provide a brief yet sensitive and comprehensive assessment of cognition in patients with MS as part of their routine clinical care.

### Relationship to Clinical and Demographic Variables

Consistent with previous research (34), longer disease duration was associated with poorer processing speed and attention. Higher EDSS scores were significantly associated with poorer working memory, executive function, processing speed, and attention. The EDSS is rated by a neurologist and is heavily reliant on walking as the main factor for quantifying disability (29). However, this measure has consistently been reported to be among the strongest correlates of cognitive impairment in MS (35), suggesting similar processes may drive both physical and cognitive disability in patients. We did not identify any statistically significant differences between the different MS disease subtypes in the proportion of patients with impairment across any of the cognitive domains. This is likely due to a lack of power to detect group differences in cognitive performance due to the relatively small number of patients with progressive forms of illness. Larger studies have reported that those MS patients with a progressive disease course exhibit more severe and widespread deficits in cognitive performance compared to those with a relapsing–remitting course (36, 37). This is perhaps unsurprising given that relapsing–remitting MS is characterized by bouts of inflammation and periods of recovery and remyelination whereas in the progressive phase, episodes of inflammation and demyelination become more infrequent with a shift toward neurodegeneration and sustained damage (38).

Cognitive reserve has been identified as a potential buffer against cognitive impairment with intellectually enriching experiences, such as high levels of education, thought to protect against MS-related cognitive decline (27, 39). We failed to identify any significant differences in the proportion of MS patients with cognitive impairment between those who left education prior to age 18 compared to those who went on to further education, though this may be attributable to a lack of variability in the data based on these categories. High GDS scores (indicative of depression) were associated with impaired processing speed, though it is difficult to determine the direction of causality in this cross-sectional association. Patients with major depressive disorder display impaired cognitive performance on CANTAB tests (23); however, cognitive dysfunction has also been shown to adversely affect social and occupational functioning and quality of life in patients with MS (7, 11, 12), which is also likely to drive depressive symptoms.

### Clinical Implications

Multiple sclerosis is a disease characterized by considerable patient heterogeneity in clinical presentation, lesion profile, and degree of cognitive dysfunction (4, 40, 41). Cognitive assessment should therefore be an important part of routine clinical care for examining the impact and progression of MS. Detection of cognitive impairment in MS is important, as it allows appropriate support to be instituted, and practical measures to overcome functional deficits to be implemented. Deficits are most commonly reported in processing speed, episodic memory, attention, and executive function among patients with MS, while other domains such as language abilities typically remain intact (4, 6). The results of this study indicate that CANTAB tasks are sensitive to impairment in each of these domains most frequently affected in MS. Though there are currently no regulatory approved pharmacological treatments for the amelioration of cognitive dysfunction in MS (42, 43), there is emerging evidence that cognitive rehabilitation interventions and physical exercise may exert pro-cognitive effects in this population and may be useful in limiting further cognitive decline (44–48).

### Strengths and Limitations

Cognition was assessed using a brief, computerized cognitive test battery that has been developed and validated by over 30 years of research (17–19), and which has previously been shown to be sensitive to cognitive deficits in patients with MS (24–26). Computerized cognitive testing helps to overcome many of the practical limitations of existing paper-and-pencil- based cognitive tasks in routine clinical care and has been shown to be well-tolerated by patients, even among very elderly individuals (49). Though the CANTAB battery used in this project was designed to assess the cognitive domains most frequently impaired in MS, there is an emerging literature suggesting that other cognitive processes, such as social cognition (5, 50), may also be adversely affected in this population. However, the “real world” impact of deficits in these abilities remain poorly understood and warrant further investigation prior to inclusion into routine cognitive assessment in clinical care (51).

Nine patients did not complete the CANTAB tests; however, no further clinical or demographic information was recorded for these individuals. It is therefore unclear whether they are missing at random or whether this introduces some bias into the current analyses. Data on the use of disease modifying therapies were also not collected at the time of the assessments, though there is currently limited evidence that these drugs have an impact on cognition (43). An additional limitation of the current study is that information relating to fatigue and daytime sleepiness was not recorded. These are common symptoms among patients with MS and may be associated with poor cognitive performance (4, 52). Depressive symptoms were assessed using the GDS, though originally developed for use in older adults, the content is generic and not age-specific (30). Published studies in both clinical and non-clinical populations have demonstrated that this is a sensitive and reliable tool for assessing depressive symptoms in adults of all ages (53–55).

### Conclusion

Results from this study confirm that cognitive impairment is common among patients with MS, that it occurs across a range of domains, and is associated with disease-related variables. CANTAB tasks provide a sensitive and practical tool for cognitive testing in MS patients as part of a holistic patient assessment. These computerized touch screen tests help to overcome many of the challenges faced when assessing cognition in clinical practice.

## Ethics Statement

As cognitive assessments were performed as part of routine clinical evaluation of patients, and clinical data was handled in an anonymized fashion, the Local Research Ethics Committee deemed that specific ethical approval was not required for this study.

## Author Contributions

SP, KM, and NV were responsible for the design of the study. FC and JB recommended suitable cognitive tests and interpreted the data. NV, SC, DL, DC, KM, and SP were all involved in patient recruitment and assessment. JC conducted the statistical analyses, interpreted the data, and drafted the manuscript. All of the authors critically reviewed and approved the manuscript prior to its submission for publication.

## Conflict of Interest Statement

JC, FC, and JB are employees of Cambridge Cognition Ltd. The remaining authors report no other conflict of interest.
